# Learning Analytics of a National Entrustable Professional Activities Platform: Cross-Sectional Study of System-Level Constraints on Advanced Entrustment in Competency-Based Medical Education

**DOI:** 10.2196/95066

**Published:** 2026-05-27

**Authors:** Jeng-Wen Chen, Rui-Bin Yu, Kai-Nan Lin, Yukiko Ono, Xuan-Bing Yu, Wei-Chung Hsu, Pa-Chun Wang, Li-Jen Liao, Chun-Hou Liao, Mingchih Chen

**Affiliations:** 1 Department of Otolaryngology–Head and Neck Surgery Cardinal Tien Hospital and College of Medicine Fu Jen Catholic University New Taipei City Taiwan; 2 Department of Otolaryngology–Head and Neck Surgery National Taiwan University Hospital Taipei Taiwan; 3 Department of Hospital Management Graduate Institute of Business Administration Fu Jen Catholic University New Taipei City Taiwan; 4 Department of Otolaryngology–Head and Neck Surgery Cardinal Tien Hospital and Fu Jen Catholic University New Taipei City Taiwan; 5 Institute of Educational Administration and Evaluation University of Taipei Taipei Taiwan; 6 Data Science Center College of Medicine Fu Jen Catholic University New Taipei City Taiwan; 7 Department of Otolaryngology, Head and Neck Surgery National Taiwan University Hospital and Children's Hospital Taipei Taiwan; 8 Sleep Center National Taiwan University Hospital Taipei Taiwan; 9 Department of Otolaryngology College of Medicine National Taiwan University Taipei Taiwan; 10 Department of Otolaryngology Cathay General Hospital Taipei Taiwan; 11 School of Medicine Fu-Jen Catholic University New Taipei City Taiwan; 12 Department of Medical Research China Medical University Hospital China Medical University Taichung Taiwan; 13 Far Eastern Memorial Hospital New Taipei City Taiwan; 14 Division of Urology Department of Surgery Cardinal Tien Hospital New Taipei City Taiwan; 15 Artificial Intelligence Development Center Fu Jen Catholic University New Taipei City Taiwan

**Keywords:** competency-based medical education, learning analytics, entrustable professional activities, clinical competency committees, workplace-based assessment, digital assessment systems

## Abstract

**Background:**

Competency-based medical education (CBME) relies on entrustable professional activities (EPAs) and Clinical Competency Committee (CCC) deliberation to support defensible decisions about trainee progression. As digital assessment platforms increasingly aggregate workplace-based assessment data across training programs, large-scale learning analytics can provide new insights into how entrustment decisions are generated and interpreted within CBME systems. However, little is known about how national assessment infrastructures influence patterns of entrustment attainment.

**Objective:**

This study examined national CCC summative entrustment decisions to identify system-level factors associated with attainment of expected supervision levels across residency training.

**Methods:**

We conducted a cross-sectional analysis of nationwide CCC entrustment data derived from the Emyway digital assessment platform used by all accredited otolaryngology–head and neck surgery residency programs in Taiwan. The dataset included 3504 summative entrustment decisions across 12 EPAs for 292 residents. Observed supervision levels were compared with prespecified targets for each training year. Logistic regression models were used to identify factors associated with attainment of expected supervision levels, including training stage, EPA sequencing, CCC review cadence, and program characteristics.

**Results:**

Median supervision levels increased across training years but did not consistently reach expected targets in later stages. Expected entrustment targets were met in 2558 of 3504 decisions (73%). Underattainment was most pronounced in senior training years, particularly R4 and R5. In multivariable analyses, delayed EPA sequencing (adjusted odds ratio 0.75, 95% CI 0.63-0.91) and lower CCC review cadence (adjusted odds ratio 0.41, 95% CI 0.28-0.61) were independently associated with lower attainment. Program-level variability in entrustment outcomes was also observed.

**Conclusions:**

Nationwide learning analytics of CCC entrustment decisions revealed a reproducible late-stage gap between expected and observed autonomy in CBME training. This pattern was associated with features of assessment system design that may be modifiable rather than trainee characteristics alone. National benchmarking of digital assessment data can therefore provide actionable insights to optimize EPA sequencing, CCC governance, and evidence generation within CBME systems.

## Introduction

Competency-based medical education (CBME) aims to ensure that progression through training reflects demonstrated clinical competence rather than time alone [1-5]. Entrustable professional activities (EPAs) [6,7] operationalize this goal by linking clinical work to supervision levels that reflect increasing autonomy [8-10]. Within programmatic assessment systems, Clinical Competency Committees (CCCs) synthesize multiple workplace-based assessments (WBAs) [11,12] to determine whether trainees can be entrusted with progressively independent clinical responsibilities [10,13-16].

As CBME implementation expands, digital assessment platforms have become central infrastructures for collecting and aggregating assessment data across training programs [5]. These platforms enable large-scale analysis of assessment patterns, creating opportunities to apply learning analytics to understand how entrustment decisions are generated within complex educational systems [17]. While prior research has examined individual EPA assessments and local CCC processes [18,19], fewer studies have analyzed national entrustment data to identify structural factors that influence progression across training programs.

Understanding these system-level dynamics is particularly important for high-stakes entrustment decisions [20]. If patterns of entrustment attainment systematically deviate from expected trajectories, this may reflect constraints [21] in the design or implementation of assessment systems rather than limitations in trainee development alone [22,23]. National digital assessment platforms, therefore, provide a unique opportunity to examine whether features of assessment architecture—such as EPA sequencing, committee review cadence, and program context—shape observed entrustment outcomes [16,24,25].

Recent scholarship has highlighted the potential of learning analytics to strengthen CCC processes and increase the value of data-informed decision-making in CBME [16,26]. Yet much of the existing literature remains focused on single institutions, local dashboards, or implementation experiences rather than national-scale analyses of summative entrustment patterns across programs. As a result, relatively little is known about how digital assessment infrastructures can be used to detect system-level bottlenecks in entrustment progression or to identify modifiable features of assessment design associated with under-attainment of expected supervision targets.

National EPA infrastructures offer a particularly useful setting for this type of analysis because they combine standardized frameworks with cross-program data collection [27]. In Taiwan, the Taiwan Society of Otorhinolaryngology-Head and Neck Surgery (ORL–HNS) has implemented a national EPA-based digital assessment system through the Emyway platform, which has accumulated more than 35,000 formative EPA records since 2022 [21,28,29]. Building on this infrastructure, a nationwide pilot in 2025 required all accredited ORL–HNS residency programs to upload CCC summative entrustment decisions for each resident and each EPA through the same electronic platform. This created a national dataset spanning programs, residents, EPAs, and training stages, providing an opportunity to examine summative entrustment decisions through a learning analytics lens [16,30].

Using nationwide CCC summative data derived from a digital EPA assessment platform, this study examined patterns of entrustment attainment across residency training. We sought to identify system-level factors associated with meeting expected supervision targets and to explore how national learning analytics can inform the design and governance of CBME assessment systems.

## Methods

### Study Design and Data Source

We conducted a national cross-sectional analysis of summative entrustment decisions recorded through a shared digital assessment platform (Emyway) supporting CBME implementation across all accredited ORL–HNS residency programs in Taiwan [28]. The platform is a cloud-based educational information system that records WBAs and CCC summative entrustment decisions across institutions. The CCC summative entrustment decisions are entered into Emyway by program administrators after CCC meetings, using standardized EPA definitions and supervision-level criteria. The platform stores these decisions in a centralized database, enabling aggregation across programs for national benchmarking and research.

In 2025, the Taiwan Society of ORL–HNS implemented a nationwide pilot requiring all programs to submit CCC summative entrustment-supervision levels for each resident and each EPA through the platform. Reporting occurred twice during the year (January and July). During each cycle, programs submitted one CCC summative entrustment level per resident-EPA combination, reflecting committee-level judgments synthesized from longitudinal WBA data. The resulting dataset comprised residents from 35 accredited training programs nationwide and reflects formal summative entrustment decisions used to determine trainee progression within the national EPA framework.

### Digital Assessment Platform

Emyway supports EPA-based programmatic assessment by enabling faculty to submit WBA records, aggregating performance data across EPAs, and providing dashboards for CCC review [28,29]. Summative entrustment decisions were recorded within the Emyway platform through structured CCC processes involving multiple faculty reviewers, with standardized EPA definitions and supervision level criteria (Level 1: observation only; Level 2: direct supervision; Level 3: indirect supervision; Level 4: independent practice; Level 5: supervise others), supporting the consistency and reliability of the recorded data. Following CCC meetings, program administrators enter final summative entrustment decisions into the platform, where the data are stored in a centralized database that supports national benchmarking and research.

### National EPA Framework

The national ORL–HNS EPA framework comprises 12 specialty-specific EPAs covering the major domains of clinical practice (Multimedia Appendix 1). Each EPA is evaluated using a 5-level entrustment-supervision scale adapted from established CBME frameworks. The framework defines expected supervision levels for each stage of training (R1-R5) (Table 1). The expected entrustment targets were defined a priori based on the national ORL–HNS EPA framework developed by the Taiwan Society of Otolaryngology–Head and Neck Surgery through expert consensus during implementation. These targets represent the intended progression of supervision levels across training years and are used operationally for programmatic assessment and benchmarking, rather than being derived from empirical validation. Summative entrustment decisions represent CCC judgments synthesized from formative EPA assessments collected within the platform.

**Table 1:** Expected entrustment-supervision levels used for summative assessment according to resident seniority.

EPA^a^ number	EPA title	Summative entrustment-supervision levels for respective resident seniority				
		R1	R2	R3	R4	R5
EPA01	(Airway) Assessing and managing patients with airway presentations	2	3	3	4	5
EPA02	(Foreign Body) Assessing and managing patients with suspicious foreign body presentations	2	3	3	4	5
EPA03	(Bleeding) Assessing and managing patients with upper aerodigestive tract bleeding presentations	2	3	3	4	5
EPA04	(Vertigo) Assessing and managing patients with vertigo	2	3	3	4	5
EPA05	(Infection) Assessing and managing patients with head and neck infections	2	3	3	4	5
EPA06^b^	(Head and Neck) Assessing and managing patients with head and neck masses (including oral cavity tumors)	2	2	3	4	5
EPA07	(Ear) Assessing and managing patients with ear and hearing diseases	2	3	3	4	5
EPA08	(Sinonasal) Assessing and managing patients with sinonasal diseases	2	3	4	4	5
EPA09	(Larynx) Assessing and managing patients with laryngopharyngeal diseases (voice/speech/language/dysphagia)	2	3	3	4	5
EPA10^b^	(Sleep Disordered Breathing) Assessing and managing patients with sleep-disordered breathing	2	2	3	4	5
EPA11^b^	(Plasty) Assessing and managing patients with facial plastic and reconstructive surgery	2	2	3	3	4
EPA12	(Oral Presentation) Case presentation	2	3	4	4	5

^a^EPA: entrustable professional activity.

^b^Delayed EPAs: EPA06, EPA10, and EPA11.

### Participants

The study population included all residents enrolled in accredited ORL–HNS residency programs in Taiwan during 2025; no additional exclusion criteria were applied. Residency training spans 5 specialty years (R1-R5), corresponding to postgraduate years PGY3-PGY7 following completion of a 2-year postgraduate general training program. Faculty participants were attending physicians who contributed workplace-based EPA assessments that informed CCC deliberations.

### Variables

CCC ratings were recorded using a five-level entrustment-supervision scale [10,31,32]. The primary outcome was dichotomized as meeting versus not meeting the expected entrustment-supervision level because the national CBME framework uses prespecified stage-based targets to monitor learner progression and determine whether residents have achieved the expected level of entrustment for each EPA (Table 1).

Independent variables represented features of the assessment system and program context. EPA sequencing was defined based on the typical order of clinical exposure within the national ORL–HNS curriculum. EPAs related to common outpatient care and emergency presentations are introduced earlier, whereas those involving subspecialty procedures, complex consultations, or advanced decision-making occur later as experience accumulates. Accordingly, early versus delayed EPAs reflect curricular design and expected learning progression rather than assessment logistics. Delayed EPAs were defined as EPAs that are typically expected to be achieved at later stages of training due to their higher complexity, requirement for advanced clinical judgment, and dependence on accumulated experience. In this study, EPA6, EPA10, and EPA11 were categorized as delayed EPAs based on expert consensus within the national specialty framework and prior curriculum design. CCC review cadence was classified as either 2 reviews per year or one review per year and was operationalized using platform-recorded submission frequency in Emyway during 2025. Programs were categorized based on whether they submitted CCC summative entrustment decisions in both scheduled cycles (January and July) or in only one cycle; this variable was derived from platform records rather than self-report. Resident-level covariates included training year (R1-R5) and sex. Program-level variables included hospital level (medical center vs nonmedical center), geographic region, and faculty size (number of attending physicians involved in resident training and assessment). Variables were selected a priori based on prior literature and theoretical considerations related to programmatic assessment systems. For factor analyses, data from the July 2025 reporting cycle were used to ensure contemporaneous assessment across EPAs.

### Statistical Analysis

Descriptive statistics were used to summarize resident, program, and assessment characteristics. Differences between assessments that met or did not meet expected entrustment targets were examined using chi-square tests. We considered potential sources of bias, including measurement variability in CCC entrustment decisions and residual confounding by program and training level; selection bias was minimized through nationwide inclusion, and remaining biases were addressed through model adjustment and sensitivity analyses. The unit of analysis was the CCC summative entrustment decision for each resident-EPA combination. Although some residents contributed decisions from more than one review cycle, each decision was made through a formal CCC process involving different faculty members and EPA-specific evidence inputs; therefore, these decisions were analyzed as distinct observations. Associations between explanatory variables and attainment of expected entrustment levels were evaluated using logistic regression models. Univariable analyses were first performed, followed by multivariable logistic regression including hospital level, faculty size, geographic region, CCC review cadence, EPA sequencing, resident training year, and sex. Results are reported as odds ratios with 95% CIs. Stratified analyses were conducted by resident training year and EPA sequencing to assess the consistency of associations across training stages. Developmental patterns in entrustment progression were examined by comparing observed CCC supervision levels with predefined training targets, summarized using medians with IQRs. All tests were 2-sided with statistical significance set at *P*<.05. Analyses were performed using SAS (version 9.4; SAS Institute, Cary, NC, USA).

### Ethical Considerations

The study was approved by the Institutional Review Board of Cardinal Tien Hospital (CTH-112-2-1-002). Participants provided electronic informed consent prior to accessing the Emyway platform by reviewing the “Training-Related Data Collection and Privacy Information” and selecting the agreement option. Participation was uncompensated. All data were deidentified prior to analysis, and access to the dataset was restricted to the research team through secure, password-protected servers.

## Results

### Progression of Entrustment-Supervision Levels Across Training Years

**Figure 1:**
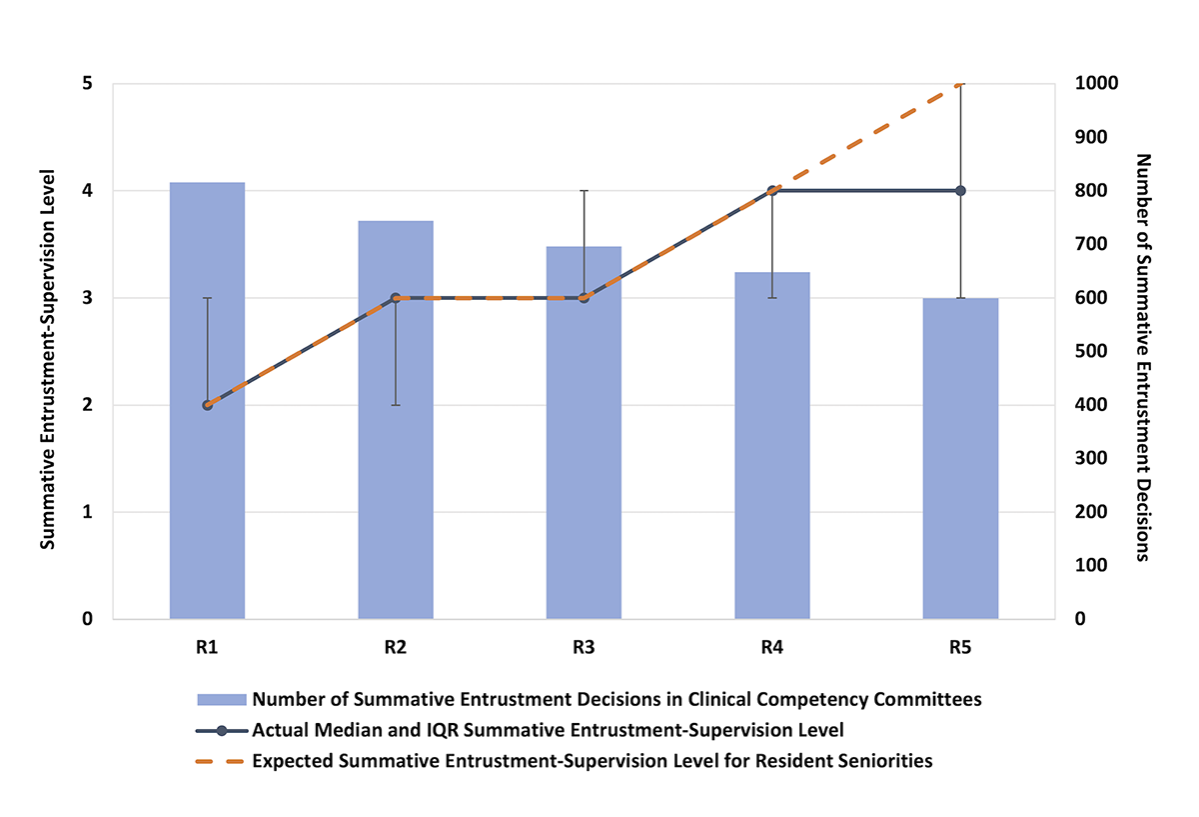
Progression of Clinical Competency Committee (CCC) summative entrustment-supervision levels across residency years in Taiwan otorhinolaryngology-head and neck surgery training programs. The solid line with error bars represents the observed median (IQR) CCC summative entrustment-supervision level by residency year, overlaid with the expected entrustment-supervision targets for each training stage (dashed line). Shaded bars (right y-axis) indicate the number of CCC summative entrustment decisions contributing to each residency year. Expected targets were prespecified according to the national framework (R1: Level 2; R2-R3: Level 3; R4: Level 4; R5: Level 5).

Observed CCC summative entrustment-supervision levels were compared with prespecified targets for each training year (Figure 1). Median (IQR) observed levels increased across training stages—from 2 (2-3) in R1, to 3 (2-3) in R2, 3 (3-4) in R3, and 4 (3-4) in both R4 and R5. Despite this upward progression, observed supervision levels did not consistently reach the expected targets in later training years.

Median observed supervision levels achieved expectations in R1 through R4 but fell below expected targets in R5. The discrepancy between expected and observed supervision levels increased with advancing training year. The largest gap was observed in R5, where the expected supervision level was Level 5, whereas the median observed level remained Level 4 (IQR 3-4).

### Characteristics by Achievement Status

The dataset included 3504 CCC summative entrustment decisions across 12 EPAs for 292 residents nationwide. Sixteen residents contributed data from only one CCC reporting cycle (July 2025), accounting for 192 observations. Overall, 2558 assessments (73%) met the expected entrustment-supervision targets, whereas 946 assessments (27%) did not. Comparisons of assessments that met versus did not meet expected targets are shown in Table 2. Nonattainment was significantly associated with larger resident quotas, northern geographic region, advanced training year, lower CCC review cadence (1 vs 2 reviews per year), and delayed EPA sequencing (all *P*<.001). Nonattainment was most frequent in senior training years, particularly R4 (20.2%) and R5 (42.2%), in programs conducting a single CCC review per year (15.8%), and in delayed EPAs (28.2%). In contrast, hospital level, municipality type, faculty size, and resident sex were not significantly associated with achievement status.

**Table 2:** Characteristics of institutions, entrustable professional activity (EPA) assessments, and participants by achievement status.

Variable				Meet expectations (n=2558), n (%)		Did not meet expectations (n=946), n (%)		*P* value	
**Program characteristics**									
	**Hospital level**								.34
		Medical center	1890 (73.9)		714 (75.5)				
		Nonmedical center	668 (26.1)		232 (24.5)				
	**Municipality type**								.35
		Special	2216 (86.6)		808 (85.4)				
		Nonspecial	342 (13.4)		138 (14.6)				
	**Resident quota**								<.001^a^
		≥3	913 (35.7)		419 (44.3)				
		2	874 (34.2)		278 (29.4)				
		1	771 (30.1)		249 (26.3)				
	**Number of faculty**								.93
		>10	1543 (60.3)		569 (60.2)				
		≤10	1015 (39.7)		377 (39.9)				
	**Geographic region**								<.001^a^
		Northern	1274 (49.8)		538 (56.9)				
		Central	638 (24.9)		238 (25.2)				
		Southern	556 (21.7)		140 (14.8)				
		Eastern	90 (3.5)		30 (3.2)				
**Resident characteristics**									
	**Seniority**								<.001^a^
		R1	701 (27.4)		115 (12.2)				
		R2	596 (23.3)		148 (15.6)				
		R3	603 (23.6)		93 (9.8)				
		R4	457 (17.9)		191 (20.2)				
		R5	201 (7.9)		399 (42.2)				
	**Sex**								.41
		Male	1786 (69.8)		674 (71.3)				
		Female	772 (30.2)		272 (28.8)				
**EPA sequencing**									
	**Planned training stage**								.007^a^
		Delayed	609 (23.8)		267 (28.2)				
		Early	1949 (76.2)		679 (71.78)				
**Assessment cadence**									
	**Two assessments**								<.001^a^
		No	43 (1.7)		149 (15.8)				
		Yes	2515 (98.3)		797 (84.2)				

^a^Statistically significant results, defined as *P*<.05.

### Factors Associated With Achieving Expected Entrustment Levels

Results of the univariable and multivariable logistic regression analyses are presented in Table 3.

In univariable analyses, training in the southern region was associated with higher odds of meeting expected entrustment targets. In contrast, larger resident quotas, delayed EPA sequencing, lower CCC review cadence, and advancing training year were associated with lower odds of attainment. Compared with R1, the likelihood of meeting expected supervision targets declined in R2, R4, and R5. Hospital level, municipality type, faculty size, and resident sex were not significantly associated with attainment.

In the multivariable model, 2 system-level variables remained independently associated with lower attainment: lower CCC review cadence (adjusted odds ratio [aOR] 0.41, 95% CI 0.28-0.61) and delayed EPA sequencing (EPA6, EPA10, and EPA11), defined as higher-complexity activities typically achieved later in training, were associated with lower entrustment attainment compared with early EPAs (aOR 0.75, 95% CI 0.63-0.91). The resident quota was excluded from the multivariable model due to strong dependency on faculty number, which led to redundancy and model instability. The faculty number was retained as it more directly reflects supervision capacity. Training in the southern region remained positively associated with attainment (aOR 1.42, 95% CI 1.12-1.79). Resident seniority also remained a strong predictor, with significantly lower attainment observed in R2, R4, and R5 relative to R1. Hospital level, faculty size, resident sex, and other geographic regions were not independently associated with attainment after adjustment.

**Table 3:** Univariable and multivariable logistic regression analyses of factors associated with achieving the expected entrustment-supervision level.

Variable				Univariable model				Multivariable model		
				OR^a^ (95% CI)		*P* value		Adjusted OR (95% CI)		*P* value
**Program characteristics**										
	**Hospital level**									
		Medical center vs Nonmedical center	0.92 (0.77-1.09)		.34		0.85 (0.66-1.09)		.21	
	**Municipality type**									
		Special vs Nonspecial	1.11 (0.89-1.37)		.35		—^b^		—	
	**Resident quota**									
		2 vs 1	1.02 (0.83-1.24)		.88		—		—	
		≥3 vs 1	0.70 (0.59-0.85)		<.001		—		—	
	**Number of faculty**									
		>10 vs ≤10	1.01 (0.87-1.17)		.93		1.11 (0.88-1.39)		.37	
	**Geographic region**									
		Central vs Northern	1.13 (0.95-1.36)		.18		0.99 (0.81-1.21)		.92	
		Southern vs Northern	1.68 (1.36-2.07)		<.001		1.42 (1.12-1.79)		.003^c^	
		Eastern vs Northern	1.27 (0.83-1.94)		.28		1.31 (0.81-2.12)		.27	
**Resident characteristics**										
	**Seniority**									
		R2 vs R1	0.66 (0.51-0.86)		.002		0.69 (0.53-0.91)		.008^c^	
		R3 vs R1	1.06 (0.79-1.43)		.68		1.10 (0.82-1.48)		.51	
		R4 vs R1	0.39 (0.30-0.51)		<.001		0.40 (0.31-0.52)		<.001^c^	
		R5 vs R1	0.08 (0.06-0.11)		<.001		0.11 (0.08-0.14)		<.001^c^	
	**Sex**									
		Male vs Female	0.93 (0.79-1.10)		.41		0.93 (0.77-1.13)		.48	
	**EPA^d^ sequencing**									
		Delayed vs Early	0.79 (0.67-0.94)		.007		0.75 (0.63-0.91)		.003^c^	
**Assessment cadence**										
	**2 CCC^e^ evaluations**									
		No vs Yes	0.09 (0.06-0.13)		<.001		0.41 (0.28-0.61)		<.001^c^	

^a^OR: odds ratio.

^b^Not applicable.

^c^Statistically significant results, defined as *P*<.05.

^d^EPA: entrustable professional activity.

^e^CCC: Clinical Competency Committee.

### Stratified Analyses

Stratified analyses suggested heterogeneity of associations across training stages and EPA sequencing (Table 4 and Table S1 in Multimedia Appendix 2).

When stratified by training year, hospital level and faculty size showed differing associations across stages. Training in a medical center was positively associated with attainment in R2 but negatively associated in R5, whereas larger faculty size showed the opposite pattern. Regional differences were most evident in R5, where attainment was higher outside the northern region. Sex was associated with attainment only in R2. Lower CCC review cadence showed the strongest negative association in R2 but was not significant in R5.

**Table 4:** Multivariable logistic regression analyses of factors associated with achieving the expected entrustment-supervision level, stratified by resident seniority.

Variable, seniority					OR^a^ (95% CI)		*P* value
**Program characteristics**							
	**Hospital level**						
		**Medical center vs Nonmedical center**					
			Overall	0.85 (0.66-1.09)		.21	
			R1	1.68 (0.91-3.11)		.09	
			R2	1.92 (1.07-3.46)		.029^b^	
			R3	0.69 (0.35-1.36)		.28	
			R4	0.64 (0.38-1.07)		.09	
			R5	0.32 (0.18-0.58)		<.001^b^	
	**Number of faculty**						
		**>10 vs** **≤** **10**					
			Overall	1.11 (0.88-1.39)		.37	
			R1	1.09 (0.61-1.94)		.78	
			R2	0.53 (0.30-0.93)		.027^b^	
			R3	1.34 (0.74-2.41)		.34	
			R4	1.14 (0.71-1.81)		.59	
			R5	2.69 (1.52-4.77)		.001^b^	
	**Geographic region**						
		**Central vs Northern**					
			Overall	0.99 (0.81-1.21)		.92	
			R1	0.79 (0.49-1.28)		.33	
			R2	1.10 (0.69-1.76)		.69	
			R3	0.86 (0.52-1.43)		.56	
			R4	0.93 (0.59-1.49)		.77	
			R5	2.26 (1.42-3.59)		.001^b^	
		**Southern vs Northern**					
			Overall	1.42 (1.12-1.79)		.003^b^	
			R1	1.44 (0.84-2.48)		.19	
			R2	1.12 (0.66-1.90)		.67	
			R3	1.50 (0.73-3.08)		.27	
			R4	1.09 (0.67-1.76)		.73	
			R5	2.73 (1.60-4.65)		<.001^b^	
		**Eastern vs Northern**					
			Overall	1.31 (0.81-2.12)		.27	
			R1	5.35 (0.68-41.98)		.11	
			R2	0.81 (0.32-2.06)		.66	
			R3	2.43 (0.29-20.59)		.42	
			R4	0.80 (0.30-2.09)		.65	
			R5	2.77 (1.07-7.15)		.036^b^	
**Resident characteristics**							
	**Sex**						
		**Male vs Female**					
			Overall	0.93 (0.77-1.13)		.48	
			R1	1.34 (0.87-2.08)		.19	
			R2	1.67 (1.02-2.74)		.043^b^	
			R3	0.83 (0.49-1.41)		.49	
			R4	0.74 (0.48-1.16)		.19	
			R5	0.78 (0.52-1.18)		.24	
	**EPA^c^ sequencing**						
		**Delayed vs Early**					
			Overall	0.75 (0.63-0.91)		.003^b^	
			R1	0.36 (0.24-0.54)		<.001^b^	
			R2	2.67 (1.57-4.55)		<.001^b^	
			R3	0.44 (0.28-0.70)		.001^b^	
			R4	0.69 (0.47-1.00)		.052	
			R5	0.91 (0.60-1.37)		.64	
**Assessment cadence**							
	**2 CCC^d^ evaluations**						
		**No vs Yes**					
			Overall	0.41 (0.28-0.61)		<.001^b^	
			R1	N/A^e^		N/A	
			R2	0.02 (0.00-0.14)		<.001^b^	
			R3	N/A		N/A	
			R4	N/A		N/A	
			R5	0.83 (0.51-1.33)		.43	

^a^OR: odds ratio.

^b^Statistically significant results, defined as *P*<.05.

^c^EPA: entrustable professional activity.

^d^CCC: Clinical Competency Committee.

^e^N/A: Estimates for R1, R3, and R4 were not reported because of insufficient sample size.

Associations between delayed EPA sequencing and attainment also varied by training year. Lower attainment was observed in R1 and R3, higher attainment in R2, and no clear association in R5. When stratified by EPA sequencing, training in the southern region remained positively associated with attainment for both early and delayed EPAs (Table S1 in Multimedia Appendix 2). Larger faculty size was associated with higher attainment only for delayed EPAs. Seniority effects differed by sequencing: for early EPAs, attainment declined progressively with advancing training year, whereas for delayed EPAs, attainment was higher in R2 but lower in R4 and R5. Lower CCC review cadence remained negatively associated with attainment across both sequencing groups.

Program-level analyses demonstrated substantial variability between training programs. Relative to the reference program, approximately half of the nonreference programs showed significantly different odds of meeting expected entrustment targets, with both higher and lower attainment observed (Tables S2 and S3 in Multimedia Appendix 2).

## Discussion

### Principal Findings

This national analysis of CCC entrustment decisions demonstrates that aggregation of WBA data alone is insufficient to ensure alignment between expected and documented trainee autonomy. Despite the synthesis of longitudinal evidence through CCC deliberation, attainment of prespecified entrustment targets declined in the most advanced stages of training, revealing a reproducible late-stage gap between expected and observed supervision levels. Importantly, this pattern was not random in distribution; 2 system-level features—delayed sequencing of complex EPAs and lower CCC review cadence—were independently associated with reduced attainment. These findings suggest that advanced entrustment decisions are influenced by both trainee capability and the architecture of the assessment systems used to generate and interpret evidence. Viewed through the lens of learning analytics, national digital assessment platforms may function as diagnostic infrastructures for CBME, revealing structural constraints on trainee progression and identifying system-level design features that are associated with how competence is documented, interpreted, and ultimately trusted.

### Seniority Gap and Late-Stage Entrustment Plateau

A central finding of this study is the persistence of a late-stage discrepancy between expected and observed entrustment levels. The expected entrustment targets were defined by expert consensus within the national EPA framework and represent intended progression rather than empirically validated thresholds; thus, the observed nonattainment rate should be interpreted relative to these consensus-based benchmarks. Although supervision levels increased progressively across training years, the median CCC entrustment level plateaued at Level 4 in the most advanced stage of training, despite an expected Level 5 target. This pattern indicates that senior residents frequently approached—but did not consistently reach—the threshold for unsupervised practice as judged by CCCs. Rather than reflecting isolated measurement variability, the consistency of this pattern across programs suggests a structural feature of the assessment system influencing how advanced competence is documented.

Several mechanisms may plausibly contribute to this phenomenon. First, the evidentiary burden required to justify Level 5 entrustment is inherently higher than that required for intermediate supervision levels [33]. CCCs typically require repeated demonstrations of safe and independent clinical performance across varied contexts before endorsing unsupervised practice [34]. When opportunities to demonstrate such autonomy are limited, evidence supporting the highest entrustment level may be less likely to accumulate even among highly competent trainees [35]. In programmatic assessment systems, this phenomenon has been described as an “evidence threshold effect,” in which higher-stakes decisions demand stronger or more consistent documentation before committees are willing to endorse them [36,37].

Second, the clinical contexts encountered by senior residents may paradoxically make documentation of autonomy more difficult. Advanced trainees often manage more complex cases or work within higher-risk environments where supervisors remain appropriately involved in decision-making [28,38]. In such situations, supervisors may exercise greater caution when documenting unsupervised practice, even when the trainee demonstrates high competence. Consequently, observed supervision levels may reflect contextual supervisory practices rather than a lack of capability. Similar patterns have been described in digital assessment systems where entrustment documentation reflects both learner performance and clinical workflow constraints [39].

Third, the timing of complex EPAs may compress the period during which evidence of independent practice can accumulate. When advanced EPAs are introduced late in training, residents may have fewer opportunities to demonstrate repeated independent performance before CCC review. This shortened observation window can limit the evidentiary base available to committees and may contribute to conservative decision thresholds. Within digital assessment environments, the delayed introduction of complex competencies has been associated with reduced opportunity for iterative feedback and progressive autonomy documentation [40].

Fourth, limited opportunities to formally supervise novice learners may partly explain the lower attainment of Level 5 entrustment. In Taiwan’s otolaryngology residency training, senior residents may guide junior residents or medical students during ward care, outpatient preparation, emergency consultations, and selected procedural settings. However, these supervisory activities are often informal, context-dependent, and not consistently documented as EPA evidence. As a result, CCCs may have insufficient direct evidence to support Level 5 entrustment, even when senior residents demonstrate high clinical competence.

Together, these mechanisms suggest that the observed plateau in senior entrustment levels reflects an interaction between trainee progression and assessment system architecture. In digital CBME infrastructures, learning analytics derived from aggregated assessment data can help reveal these structural dynamics by identifying patterns of progression that are not readily visible within individual programs [5].

### Influence of CCC Review Cadence on Entrustment Outcomes

Another important finding of this study is the association between CCC review cadence and attainment of expected entrustment levels. Programs conducting only a single CCC review within an academic year showed significantly lower odds of meeting expected supervision targets compared with programs conducting 2 reviews annually. Because residents within these programs accumulate comparable numbers of EPAs, the difference is unlikely to reflect assessment volume alone. Instead, CCC review cadence likely influences how evidence is synthesized and interpreted over time. More frequent committee reviews allow CCC members to examine developmental trajectories across multiple time points, confirm whether performance patterns are stable, and provide feedback that can guide targeted clinical exposure or documentation practices. Earlier feedback cycles also enable trainees and supervisors to address evidentiary gaps before final summative decisions are made. In contrast, when only a single review occurs annually, the evidentiary window is compressed, and recent or atypical events may disproportionately influence committee judgments.

Digital assessment platforms have increasingly enabled longitudinal visualization of learner performance through dashboards and aggregated data displays, allowing committees to review developmental trajectories rather than isolated observations [41]. These tools have been proposed as mechanisms to strengthen CCC deliberation and support data-informed decision-making in CBME [5]. From a systems perspective, CCC review cadence therefore represents a modifiable governance feature within national CBME infrastructures. When combined with digital assessment platforms capable of aggregating longitudinal evidence, justified review cycles may improve the interpretability of assessment data and reduce uncertainty surrounding advanced entrustment decisions.

### Reversal Pattern in R2 Entrustment Attainment

An additional notable finding was the reversal pattern observed in R2, where attainment appeared higher for delayed EPAs but lower for early EPAs compared with R1. One possible explanation is that delayed EPAs in R2 are encountered in more structured and supervised contexts, often involving selected cases with closer faculty guidance. In contrast, early EPAs are performed more frequently in routine clinical settings, where expectations for independent performance may be higher, and variability in workload and assessment opportunities may be greater. This difference in context may contribute to the observed pattern. However, this interpretation remains speculative and warrants further investigation using longitudinal and WBA-level data.

### Program-Level Variation in Entrustment Decisions

Substantial variability in attainment rates between programs was also observed in this study. Such variability indicates that CCC entrustment outcomes reflect not only resident performance but also local implementation practices and institutional contexts [42]. Differences in case mix, supervisory structures, and clinical exposure may legitimately influence how autonomy is granted within different training environments [43].

However, wide variation in summative entrustment decisions may also arise from differences in CCC calibration, documentation practices, or local thresholds for endorsing unsupervised practice [44]. In digital CBME systems, these variations can become visible when assessment data from multiple institutions are aggregated and analyzed collectively. National learning analytics infrastructures, therefore, provide an opportunity to distinguish contextually appropriate variation from potential inconsistency in assessment practices [40,45,46].

National benchmarking of entrustment outcomes may help training programs evaluate whether local practices align with broader system patterns. When combined with faculty development and CCC calibration initiatives, such benchmarking can strengthen the fairness and comparability of high-stakes progression decisions while preserving necessary contextual flexibility.

### Regional Variation in Entrustment Attainment

Programs in the southern region consistently demonstrated higher entrustment attainment across analyses. One possible explanation is regional variation in clinical exposure, supervisory practices, or CCC calibration, which may influence how entrustment decisions are documented. Differences in case mix, faculty-resident interaction patterns, or assessment culture may also contribute. However, as these factors were not directly measured, this finding should be interpreted cautiously. Further qualitative and mixed methods studies are needed to clarify the mechanisms underlying regional variation.

### Implications for Digital CBME Systems

The findings of this study highlight the growing role of digital assessment infrastructures in shaping CBME implementation. Historically, assessment systems primarily served to document trainee performance within individual programs. Increasingly, however, national digital platforms enable aggregation and analysis of assessment data across institutions, creating opportunities for large-scale learning analytics [46-48].

Such systems allow educational leaders to examine patterns of trainee progression, identify structural constraints within training pathways, and evaluate how design features of assessment systems influence entrustment decisions. When interpreted alongside programmatic assessment theory, these analytics can guide targeted adjustments to EPA sequencing, CCC review cadence, and evidence sampling strategies.

In this sense, national digital assessment platforms function not only as repositories of assessment data but also as diagnostic infrastructures for CBME governance. By enabling cross-program benchmarking and longitudinal data analysis, these systems support more transparent and defensible progression decisions while providing actionable insights for improving training system design.

### Limitations

Several limitations should be considered when interpreting these findings. First, the cross-sectional design precludes causal inference and does not capture individual longitudinal trajectories of entrustment progression. Although the nationwide dataset provides a comprehensive system-level snapshot of CCC decisions, future studies using longitudinal data across multiple training cycles could more precisely examine developmental patterns in entrustment. Second, several contextual variables that may influence CCC judgments were not available in the analytic dataset, including variation in underlying WBA volume, quality, distribution, observed patterns, rotation-specific clinical exposure, case complexity, rater composition, and institutional autonomy policies. These factors may contribute to residual variability in entrustment outcomes across programs. Third, CCC decisions are inherently context dependent, reflecting both trainee performance and local supervisory practices. While such contextual variation may limit strict comparability across institutions, it is an expected feature of programmatic assessment systems and can provide meaningful information for national benchmarking and quality improvement. Fourth, although each CCC summative entrustment decision was treated as a distinct observation, some residents contributed multiple decisions across EPAs or review cycles. Residual within-resident correlation may therefore remain, and future analyses using multilevel or clustered models could further clarify resident- and program-level sources of variation. Moreover, while this binary outcome aligns with CCC progression decisions, it reduces the informational richness of the ordinal entrustment scale and may obscure smaller differences between adjacent supervision levels. Future studies using ordinal or longitudinal analytic approaches may provide a more granular understanding of entrustment progression. Additionally, the generalizability of these findings to other CBME contexts, even those with structured EPA frameworks and centralized assessment systems, remains uncertain and requires further study to confirm. Finally, this study examined summative entrustment decisions without linking them to downstream clinical outcomes. Future research integrating digital assessment data with measures of independent clinical performance and patient safety outcomes would further strengthen the evidence base supporting entrustment decisions within CBME.

### Conclusions

Using nationwide CCC entrustment data derived from a digital assessment platform, this study demonstrates that under-attainment of expected supervision targets in CBME is not random but systematically associated with structural features of assessment system design. Under-attainment clustered in advanced training stages, in late-sequenced EPAs, and in programs with lower CCC review cadence, suggesting the presence of a seniority gap within current assessment architectures. These findings indicate that documented entrustment outcomes reflect not only trainee capability but also how assessment evidence is generated, aggregated, and interpreted within programmatic assessment systems. National benchmarking of CCC decisions, enabled by shared digital assessment infrastructures, can therefore provide actionable learning analytics to identify structural constraints on trainee progression and inform system-level improvements in EPA sequencing, CCC governance, and evidence sampling. In this way, national digital assessment infrastructures can evolve from data repositories into strategic learning analytics systems that support continuous improvement of competency-based training.

## Appendix

Multimedia Appendix 1 Taiwan Society of Otorhinolaryngology–Head and Neck Surgery Entrustable Professional Activities Assessment Framework for Resident Physician Training.

Multimedia Appendix 2 Multivariable logistic regression analyses of factors associated with achieving the expected entrustment–supervision level, stratified by entrustable professional activity (EPA) sequencing and program-level fixed-effect estimates from logistic regression (maximum likelihood estimates) comparing each residency training program (HospID) with the reference program (H01) for attainment of the expected Clinical Competency Committee (CCC) summative entrustment–supervision level. Data on the program-level odds ratios (ORs) and Wald 95% CIs for attainment of the expected CCC summative entrustment–supervision level, comparing each training program (HospID) with the reference program (H01).

## Abbreviations

aOR: adjusted odds ratio
CBME: competency-based medical education
CCC: Clinical Competency Committee
EPA: entrustable professional activity
ORL–HNS: Otorhinolaryngology-Head and Neck Surgery
WBA: workplace-based assessment

## Data Availability

Data were obtained from the Joint Commission of Taiwan and the Taiwan Society of Otorhinolaryngology-Head and Neck Surgery Emyway database with permission for use. The datasets used and analyzed during this study are available from the corresponding author on reasonable request.
