# Supplementary material for: Learning Analytics of a National Entrustable Professional Activities Platform: Cross-Sectional Study of System-Level Constraints on Advanced Entrustment in Competency-Based Medical Education
Source: JMIR Med Educ. 2026 May 27;12:e95066. doi: 10.2196/95066 (PMC13254506; doi:10.2196/95066)
Supplement: Multimedia Appendix 2 [file mededu_v12i1e95066_app2.docx]

**Multimedia Appendix 2**

**Supplementary Table 1.** Multivariable logistic regression analyses of factors associated with achieving the expected entrustment–supervision level, stratified by EPA sequencing.

| Variable |  | EPA sequencing | OR (95%CI) | *P* value |
| --- | --- | --- | --- | --- |
| Program characteristics | |  |  |  |
| Hospital level | Medical center vs Non-medical center | Overall | 0.85 (0.66–1.09) | .209 |
|  |  | Early | 0.87 (0.64–1.17) | .349 |
|  |  | Delayed | 0.80 (0.50–1.29) | .367 |
| Number of faculty | >10 vs ≦10 | Overall | 1.11 (0.88–1.39) | .373 |
|  |  | Early | 0.95 (0.72–1.24) | .681 |
|  |  | Delayed | 1.70 (1.10–2.62) | **.016** |
| Geographic Region | Central vs Northern | Overall | 0.99 (0.81–1.21) | .918 |
|  |  | Early | 1.10 (0.86–1.40) | .465 |
|  |  | Delayed | 0.76 (0.52–1.11) | .158 |
|  | Southern vs Northern | Overall | 1.42 (1.12–1.79) | **.003** |
|  |  | Early | 1.33 (1.01–1.75) | **.039** |
|  |  | Delayed | 1.71 (1.08–2.72) | **.023** |
|  | Eastern vs Northern | Overall | 1.31 (0.81–2.12) | .265 |
|  |  | Early | 1.03 (0.60–1.78) | .906 |
|  |  | Delayed | 2.74 (0.98–7.65) | .055 |
| Resident characteristics | |  |  |  |
| Seniority | R2 vs R1 | Overall | 0.69 (0.53–0.91) | **.008** |
|  |  | Early | 0.42 (0.31–0.59) | **<.001** |
|  |  | Delayed | 2.83 (1.60–5.03) | **<.001** |
|  | R3 vs R1 | Overall | 1.10 (0.82–1.48) | .514 |
|  |  | Early | 1.02 (0.69–1.48) | .940 |
|  |  | Delayed | 1.29 (0.79–2.11) | .304 |
|  | R4 vs R1 | Overall | 0.40 (0.31–0.52) | **<.001** |
|  |  | Early | 0.32 (0.23–0.45) | **<.001** |
|  |  | Delayed | 0.59 (0.38–0.94) | **.027** |
|  | R5 vs R1 | Overall | 0.11 (0.08–0.14) | **<.001** |
|  |  | Early | 0.08 (0.06–0.11) | **<.001** |
|  |  | Delayed | 0.19 (0.11–0.31) | **<.001** |
| Sex | Male vs Female | Overall | 0.93 (0.77–1.13) | .476 |
|  |  | Early | 0.86 (0.69–1.08) | .186 |
|  |  | Delayed | 1.19 (0.84–1.70) | .331 |
| Assessment cadence | |  |  |  |
| 2 CCC evaluations | No vs Yes | Overall | 0.41 (0.28–0.61) | **<.001** |
|  |  | Early | 0.40 (0.25–0.64) | **<.001** |
|  |  | Delayed | 0.42 (0.19–0.96) | **.040** |

**Supplementary Table 2.** Program-level fixed-effect estimates from logistic regression (maximum likelihood estimates) comparing each residency training program (HospID) with the reference program (H01) for attainment of the expected CCC summative entrustment–supervision level.

| **Analysis of Maximum Likelihood Estimates** | | | | | | | |
| --- | --- | --- | --- | --- | --- | --- | --- |
| **Parameter** |  | **DF** | **Estimate** | **Standard**  **Error** | **Wald**  **Chi-Square** | **Pr > ChiSq** |  |
| Intercept |  | 1 | 1.1896 | 0.3052 | 15.1889 | **<.001** |  |
| HospID | H02 | 1 | -0.7667 | 0.3326 | 5.3157 | **.021** |  |
| HospID | H03 | 1 | 1.5919 | 0.4948 | 10.3487 | **.001** |  |
| HospID | H04 | 1 | -0.2616 | 0.3372 | 0.6019 | .438 |  |
| HospID | H05 | 1 | -0.8987 | 0.3404 | 6.9702 | **.008** |  |
| HospID | H06 | 1 | 0.9183 | 0.4236 | 4.6995 | **.030** |  |
| HospID | H07 | 1 | 0.6822 | 0.4872 | 1.9605 | .162 |  |
| HospID | H08 | 1 | -0.4964 | 0.4671 | 1.1296 | .288 |  |
| HospID | H09 | 1 | 2.3657 | 1.0591 | 4.9894 | **.026** |  |
| HospID | H10 | 1 | -0.3423 | 0.4154 | 0.6791 | .410 |  |
| HospID | H11 | 1 | -1.1229 | 0.3999 | 7.8851 | **.005** |  |
| HospID | H12 | 1 | -0.178 | 0.3685 | 0.2333 | .629 |  |
| HospID | H13 | 1 | -0.4964 | 0.4101 | 1.4655 | .226 |  |
| HospID | H14 | 1 | -0.643 | 0.4061 | 2.507 | .113 |  |
| HospID | H15 | 1 | -0.4964 | 0.4101 | 1.4655 | .226 |  |
| HospID | H16 | 1 | -0.5736 | 0.3293 | 3.0347 | .082 |  |
| HospID | H17 | 1 | 0.8348 | 0.4172 | 4.0044 | **.045** |  |
| HospID | H18 | 1 | 0.6822 | 0.4066 | 2.8159 | .093 |  |
| HospID | H19 | 1 | -0.8531 | 0.3688 | 5.3505 | **.021** |  |
| HospID | H20 | 1 | -0.3423 | 0.4154 | 0.6791 | .410 |  |
| HospID | H21 | 1 | -0.091 | 0.4267 | 0.0455 | .831 |  |
| HospID | H22 | 1 | -0.3023 | 0.3789 | 0.6364 | .425 |  |
| HospID | H23 | 1 | -0.8149 | 0.3352 | 5.9108 | **.015** |  |
| HospID | H24 | 1 | -0.7376 | 0.4583 | 2.5901 | .108 |  |
| HospID | H25 | 1 | 1.4057 | 0.5551 | 6.4134 | **.011** |  |
| HospID | H26 | 1 | -0.9889 | 0.3561 | 7.7102 | **.006** |  |
| HospID | H27 | 1 | 0.3043 | 0.4522 | 0.453 | .501 |  |
| HospID | H28 | 1 | 2.1777 | 0.7813 | 7.7693 | **.005** |  |
| HospID | H29 | 1 | 0.9183 | 0.4236 | 4.6995 | **.030** |  |
| HospID | H30 | 1 | 1.2083 | 0.429 | 7.9316 | **.005** |  |
| HospID | H31 | 1 | -0.1992 | 0.3582 | 0.3092 | .578 |  |
| HospID | H32 | 1 | 0.2768 | 0.4795 | 0.3331 | .564 |  |
| HospID | H33 | 1 | -0.9213 | 0.4013 | 5.2709 | **.022** |  |
| HospID | H34 | 1 | 1.4495 | 0.6009 | 5.8195 | **.016** |  |
| HospID | H35 | 1 | 1.2083 | 1.0882 | 1.233 | .267 |  |

**Supplementary Table 3.** Program-level odds ratios (ORs) and Wald 95% confidence intervals for attainment of the expected CCC summative entrustment–supervision level, comparing each training program (HospID) with the reference program (H01).

| **Odds Ratio Estimates and Wald Confidence Intervals** | | | | | |
| --- | --- | --- | --- | --- | --- |
| **Effect** | **Unit** | **Estimate** | | **95% Confidence Limits** | |
| HospID H02 vs H01 | 1 | 0.465 | 0.242 | | 0.891 |
| HospID H03 vs H01 | 1 | 4.913 | 1.863 | | 12.959 |
| HospID H04 vs H01 | 1 | 0.77 | 0.398 | | 1.491 |
| HospID H05 vs H01 | 1 | 0.407 | 0.209 | | 0.793 |
| HospID H06 vs H01 | 1 | 2.505 | 1.092 | | 5.746 |
| HospID H07 vs H01 | 1 | 1.978 | 0.761 | | 5.141 |
| HospID H08 vs H01 | 1 | 0.609 | 0.244 | | 1.52 |
| HospID H09 vs H01 | 1 | 10.652 | 1.336 | | 84.908 |
| HospID H10 vs H01 | 1 | 0.71 | 0.315 | | 1.603 |
| HospID H11 vs H01 | 1 | 0.325 | 0.149 | | 0.712 |
| HospID H12 vs H01 | 1 | 0.837 | 0.406 | | 1.723 |
| HospID H13 vs H01 | 1 | 0.609 | 0.272 | | 1.36 |
| HospID H14 vs H01 | 1 | 0.526 | 0.237 | | 1.165 |
| HospID H15 vs H01 | 1 | 0.609 | 0.272 | | 1.36 |
| HospID H16 vs H01 | 1 | 0.563 | 0.296 | | 1.074 |
| HospID H17 vs H01 | 1 | 2.304 | 1.017 | | 5.22 |
| HospID H18 vs H01 | 1 | 1.978 | 0.892 | | 4.389 |
| HospID H19 vs H01 | 1 | 0.426 | 0.207 | | 0.878 |
| HospID H20 vs H01 | 1 | 0.71 | 0.315 | | 1.603 |
| HospID H21 vs H01 | 1 | 0.913 | 0.396 | | 2.107 |
| HospID H22 vs H01 | 1 | 0.739 | 0.352 | | 1.553 |
| HospID H23 vs H01 | 1 | 0.443 | 0.23 | | 0.854 |
| HospID H24 vs H01 | 1 | 0.478 | 0.195 | | 1.174 |
| HospID H25 vs H01 | 1 | 4.078 | 1.374 | | 12.104 |
| HospID H26 vs H01 | 1 | 0.372 | 0.185 | | 0.748 |
| HospID H27 vs H01 | 1 | 1.356 | 0.559 | | 3.289 |
| HospID H28 vs H01 | 1 | 8.826 | 1.909 | | 40.813 |
| HospID H29 vs H01 | 1 | 2.505 | 1.092 | | 5.746 |
| HospID H30 vs H01 | 1 | 3.348 | 1.444 | | 7.762 |
| HospID H31 vs H01 | 1 | 0.819 | 0.406 | | 1.654 |
| HospID H32 vs H01 | 1 | 1.319 | 0.515 | | 3.376 |
| HospID H33 vs H01 | 1 | 0.398 | 0.181 | | 0.874 |
| HospID H34 vs H01 | 1 | 4.261 | 1.312 | | 13.834 |
| HospID H35 vs H01 | 1 | 3.348 | 0.397 | | 28.249 |
